# Prevalence study of mental disorders in an Italian region. Preliminary report

**DOI:** 10.1186/s12888-022-04401-4

**Published:** 2023-01-05

**Authors:** Caterina Silvestri, Barbara Carpita, Emanuele Cassioli, Marco Lazzeretti, Eleonora Rossi, Valentina Messina, Giovanni Castellini, Valdo Ricca, Liliana Dell’Osso, Simone Bolognesi, Andrea Fagiolini, Fabio Voller, Mental Disorders Study group

**Affiliations:** 1 Regional Health Agency of Tuscany, Epidemiology Observatory, 50100 Florence, Italy; 2 Department of Clinical and Experimental Medicine, University of Pisa, 56100 Pisa, Italy; 3 Psychiatry Unit, Department of Health Sciences, Florence University, 50100 Florence, Italy; 4 Department of Molecular and Development Medicine, University of Siena, 53100 Siena, Italy

**Keywords:** Epidemiology, Psychiatric disorders, Panic disorder, Mood disorders

## Abstract

**Background:**

Mental disorders are a major public health problem. However, over the last few years, there have been few studies aimed at evaluating their diffusion. Therefore, this study aimed at evaluating: the prevalence of the most frequent psychiatric disorders in the general population residing in Tuscany using a clinical scale administered by trainee in psychiatry.

**Methods:**

The study was carried out on a representative sample of the general population aged > 18 years, randomly extracted from the register of patients in the Tuscany region, adopting a proportional sampling method stratified by gender, age group and Local Health Units (LHU). Each person was contacted by letter followed by a phone call from an operator who makes an appointment with the trainee in psychiatry. The diagnostic interview conducted was the Mini-International Neuropsychiatric Interview (MINI). Point and lifetime prevalence by gender and age group were calculated. Differences and associations were considered statistically significant if their *p*-values were less than 0.05.

**Results:**

Of the 408 people involved, 390 people were enrolled (of which 52.6% female). The 28.5% of the sample had been affected by a psychiatric disorder during their lifetime.

In their lifetime, the most represented psychiatric disorders were major depressive episode (20.4%), major depressive disorder (17.0%) and panic disorder (10.3%), more frequent in the female than the male group. Current conditions were predominantly major depressive episode (3.1%) and agoraphobia (2.8%). A 5.9% rate of current suicidal ideation was also found.

**Conclusions:**

In the general population, 28.5% of people reported a psychiatric disorder during their lifetime. This prevalence is considerably higher than that reported in a previous study carried out in central Italy.

**Supplementary Information:**

The online version contains supplementary material available at 10.1186/s12888-022-04401-4.

## Background

According to the Constitution of the World Health Organization (WHO) [1], mental health it is “a state of complete physical, mental and social well-being and not merely the absence of disease or infirmity.” The prevalence of mental disorders (30–40% of the population) is greater than other medical conditions such as coronary diseases or neoplasia [2]. The importance of mental disorders for public health has been underestimated, even though about 14% of the global burden of disease has been attributed to neuropsychiatric disorders (such as depression, alcohol-use and substance-use disorders, and psychoses) [3]. If on the one hand, it has been estimated that the cumulative economic loss due to mental illness equates to that of cardiovascular diseases and it is even higher than cancer, diabetes and respiratory diseases [4], on the other hand, according to the Italian Ministry of Health, the mental health sector gets allocated about 3% of national health expenditure [5]. In the European context, Italy’s investment in the mental health sector is far below other countries such as Germany, France and the United Kingdom which allocate an average of 10–12% of their national health expenditure [6, 7].

The burden of mental disorders is likely to have been underestimated because of the lack of reliable estimates of the prevalence of specific disorders, as well as the connectedness between mental illness and other health conditions. For example, a recent Italian study [8] found an incidence rate of depressive and bipolar disorders of 53.61 and 1.5 per 10,000 person-years, respectively, with a strong association between these disorders and several somatic conditions, such as migraine, irritable bowel syndrome, and pelvic inflammation.

Estimation of the prevalence of the most frequent mental disorders is crucial to plan prevention programs, and the correct allocation of resources to strengthen health-care systems. However, clinical samples are not an adequate source of information, given that only a minority of people suffering from mental disorders are referred to a specialist. On the other hand, epidemiological surveys performed on the general population are often based on methods that are distant from those employed in clinical practice [9].

To date, only two investigations previously addressed prevalence of mental disorders in Italy, adopting interviewers and instruments which were typically clinical, in samples drawn from the general population. The first one was the Sesto Fiorentino study, performed in a small municipality in the centre of Italy, which estimated a lifetime prevalence of any mental disorder as 15.7% (13.5–17.8) in men, and 31.7% (29.1–34.2) in women [10]. The second study was the European Study of the Epidemiology of Mental Disorders (ESEMeD), a part of the WHO World Mental Health Survey Initiative (ESEMeD-WMH). ESEMed was a cross-sectional, face-to-face, household survey of probability samples of the adult population of six European countries, including Belgium, France, Germany, Italy, the Netherlands and Spain [11]. The study reported a lifetime prevalence of 11% for any mood disorder, 10.3% for any anxiety disorder and 1.3% for any alcohol disorder. Women were twice as likely as men to report a mood disorder and four times as likely as men to report an anxiety disorder, while men were twice as likely as women to report an alcohol disorder.

Since these two studies, no other significant surveys have been performed in almost the past 20 years in Italy. Nevertheless, during this period dramatic changes occurred in European and Italian societies (including a severe economic crisis in 2008 and the COVID-19 pandemic in 2020–2021), which are supposed to have challenged the global mental health in our countries.

## Methods

### Population sample recruitment and study design

The study was carried out on a representative sample of the general population aged > 18 years, residing in Tuscany, and registered in the health register up to 1st January 2019.

As of 1st January 2019, in Tuscany the resident population aged > 18 years was 3,146,896 with an average age of 53.6 years. Of these, 47.7% were male. 38.4% of the population aged > 9 had a secondary school diploma or a professional qualification.

A proportional sampling method stratified by gender, age group (18–29; 30–44; 45–59; 60–74; 75 and +) and Local Health Units (LHU) was adopted.

The formula we used to calculate the sample size is 1.96 ^ 2 * P * (1-P) / E ^ 2 where P is the expected lifetime prevalence of mental health disorders (18% from previous studies) (10) and E is the expected error of the confidence interval estimate (3.5%). According to this formula, the number of subjects to be screened was 450.

The LHU extracted from the patient list, for each stratum, several respondents equal to that indicated and many potential replacements with a ratio of 1:4. The substitutes belonged to the same gender, age and LHU stratum as the respondent. The entire sample consisted of 1800 people.

Participants were selected and recruited from the general population from the patient list, who were then sent a letter to invite their participation in the study by telephone. The methodology and purpose of the study were specified in this letter.

This letter was followed by a telephone call from a specially trained operator who, in case of acceptance, arranged for an appointment with the trainee in psychiatry. The day before the appointment, the person was contacted again to confirm the appointment. In case of refusal (or unavailability) of the respondents, the recruiter made a substitution scrolling through the list.

**Fig. 1:**
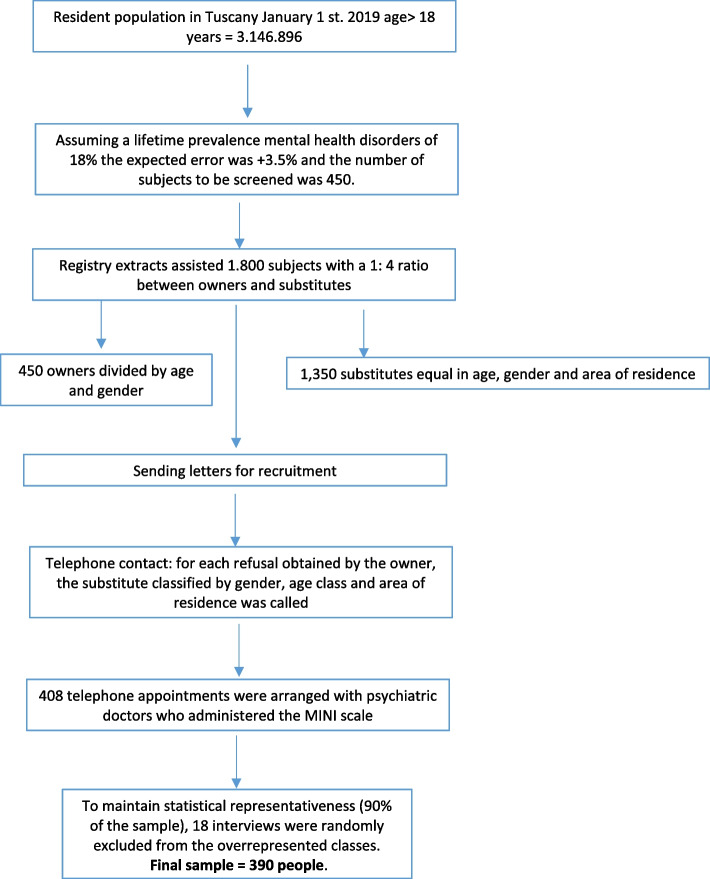
Enrollment process flowchart

The original sample defined in the survey’s protocol was 450 people but due to difficulties in contacting the individuals during the SARS-CoV-2 pandemic, 408 interviews were conducted. The subjects that refused to participate in the study were 1.392. At that point, we reached a balanced deal with the sample statistical representation and the interviews available, using 90% of the theoretical sample (400 people). We also decided to maintain the regional representation of our sample; it was possible for gender and age groups but not for the LHU because the majority of missing interviews were from one of them. Of the 408 interviews conducted, we randomly discarded 18 interviews from the overrepresented classes and reached the composition of the final sample, in which 390 people were enrolled (Fig. 1). The mean age of people enrolled compared to people that refused to participate was 52.9 years vs 49 years, with no statistically significant differences between the two cohorts. The prevalence of males in the enrolled people and that of the people who refused was 47.4 and 46.6%, respectively, with no difference between the two groups.

The interviews were carried out from 15 February 2020 to 15 July 2020.The study was approved by the Ethics Committee of Tuscany (Protocol 2019/15147_oss).

The project was carried out in accordance with the Declaration of Helsinki and its later amendments. All participants were enrolled only after providing written informed consent.

#### Eligible criteria and data collection

Information was collected on age, gender and educational level. Education level was based on current educational attainment and divided into four classes: no qualification (< 5 years); low (> = 5 and 8 years); medium (> 8 and 13 years); high (> 13 years).

Literacy in the Italian language was assessed using two categories: mainly spoken language, and understanding the Italian language. Those who did not understand the Italian language were excluded from the sample.

### Diagnostic interview

The MINI version 7.0.2 for Diagnostic and Statistical Manual of mental disorders V (DSM-5 - Italian Version of 16 Jan 2018 – Mapi. ID060037) is a short, structured diagnostic interview for DSM-5 and International Classification of Diseases 10 (ICD-10) psychiatric disorders, which evaluates the 17 most common mental health disorders. The MINI has similar reliability and validity properties to the Structured Clinical Interview for DSM-III-R - Patient (SCID-P) and the Composite International Diagnostic Interview (CIDI) [12], but it can be administered in a shorter period (mean 18.7 ± 11.6 minutes, median 15 minutes) than the above-referenced instruments.

Our study included all modules the MINI interview.

The MINI has been validated for the assessment of the presence of these disorders at the time of the interview. It also provides algorithms for obtaining a diagnosis of mood disorder (Major Depressive Disorder, Bipolar I Disorder, Bipolar II Disorder) based on the history of mood episodes. In addition, the interview also evaluates the lifetime presence of the following disorders and conditions: depression, manic and hypomanic episodes, major depressive disorder, bipolar I disorder, bipolar II disorder, panic disorder, psychotic disorders, antisocial personality disorder. Consequently, both lifetime and point prevalences were reported for these pathologies, while for all others only point prevalences were reported. For the sake of completeness, the prevalence of mood episodes (hypomanic, manic and depressive) was also reported, regardless of diagnosis.

The MINI was conducted over the telephone [13] by trainee in psychiatry within 1 week after the first contact. Psychiatry residents underwent specific training for the administration of the MINI scale.

### Statistical analyses

#### Distribution of frequency (number and percentage) of the enrolled population divided by gender, age group and educational level

Point and lifetime prevalence and 95% confidence interval (CI) for every diagnosis was reported by gender and age group. CIs were calculated with the Wald method based on a normal approximation formula using zero as the lower boundary of the interval if it was a negative value.

Two proportion Z-tests for independent populations were performed to evaluate differences in the prevalence of diagnosis by gender. Chi-square (χ^2^) tests or Fisher’s exact test for small size samples were carried out as appropriate in identifying an association between demographic factors such as age, class and educational level with diagnosis. Differences and associations were considered statistically significant if their *p*-values were less than 0.05, rejecting the null hypothesis.

All the analyses were done using Stata Statistical Software [14].

## Results

Concerning the complete 90% sample of 400 people (*n* = 390), we had under-coverage for the following classes: age 18–29 for men and over age 75 for both genders.

Females comprised 52.6% (205 individuals) of the entire sample (n = 390). Analysing the population by age group, the sample had the following distribution: 46 individuals (21 males and 25 females) were aged 18–29, 90 (45 males and 45 females) were aged 30–44, 111 (54 males and 57 females) were aged 45–59, 86 (41 males and 45 females) were aged 60–74, 57 (24 males and 33 females), (as shown in Table 1).

**Table 1:** Distribution of the sample by gender and age group

	Age Groups											
	19–29		30–44		45–59		60–74		75+		Totale	
Gender	**N**	**%**	**N**	**%**	**N**	**%**	**N**	**%**	**N**	**%**	**N**	**%**
Males	21	**11,4**	45	**24,3**	54	**29,2**	41	**22,2**	24	**13,0**	185	**100**
Females	25	**13,5**	45	**24,3**	57	**30,8**	45	**24,3**	33	**17,8**	205	**100**
Total	46	**24,9**	90	**48,6**	111	**60,0**	86	**46,5**	57	**30,8**	390	**100**

%: percentage

*N* number

The sample did not show a different distribution of educational level in the population groups defined by gender (F = 2.88; *p*-value = 0.237). The information was known just for 314 people (151 males and 163 females), distributed as follows: 46 subjects (14.6% of the sample; 18 males and 28 females) with low grade, 190 (60.5%; 98 males and 92 females) with medium grade and 78 (24.8%; 35 males and 43 females) with high grade (as shown in the supplementary Table 1).

Overall, 34.6% of the surveyed sample suffered from at least one mental disorder in their life (lifetime or current). Despite the gender difference (females: 39%; males: 29.7%), no statistical significance was shown (*p*-value = 0.054). Following the scoring algorithm provided by the M.I.N.I., the results were divided according to the period of time in which the disorder occurred (lifetime or current disorder). *The most frequent lifetime* mental disorders were major depressive episode (20.4%), major depressive disorder (17%), panic disorder (10.3%), manic episode (3.9%) and bipolar I disorder (3.9%). The prevalence observed in the female group compared to the male group for the major depressive episode, 25.5% versus 14.7% (*p*-value = 0.008) and the major depressive disorder (21.1% versus 12.5%; p-value = 0.024) was statistically significant (as shown in Table 2).

**Table 2:** Lifetime prevalence (%) of DSM-V psychiatric disorders by gender

Psychiatric disorders	Males		Females		Total			
	Lifetime prevalence	CI 95%	Lifetime prevalence	CI 95%	Lifetime prevalence	CI 95%	*Z*-test	*P*-value
Major depressive episode	14.7	[9.6–19.8]	25.5	[19.5–31.5]	20.4	[16.6–24.7]	−2.65	0.01
Major depressive disorder (DDM)	12.5	[7.7–17.3]	21.1	[15.5–26.7]	17.0	[13.6–21.1]	−2.25	0.02
Manic episode	3.3	[0.7–5.9]	4.5	[1.6–7.3]	3.9	[2.4–6.4]	−0.59	0.56
Hypomanic episode	1.1	[0.0–2.7]	1.0	[0.0–2.5]	1.1	[0.4–2.8]	0.09	0.92
Panic disorder	8.1	[4.2–12.0]	12.3	[7.8–16.8]	10.3	[7.6–13.7]	−1.34	0.18
Psychotic disorder	0.0	–	0.0	–	0.0	–	–	–
Antisocial personality disorder	1.1	[0.0–2.6]	0.5	[0.0–1.5]	0.8	[0.3–2.4]	0.67	0.50
Bipolar I disorder	3.3	[0.7–5.9]	4.5	[1.6–7.3]	3.9	[2.4–6.4]	−0.59	0.56
Bipolar II disorder	0.0	–	0.5	[0.0–1.4]	0.3	[0.0–1.8]	−0.95	0.34
At least one of the listed	23.2	[17.2–29.3]	33.2	[26.7–39.6]	28.5	[24.2–33.2]	−2.17	0.03

Differences and associations were considered statistically significant if their p-values were less than 0.05

%: percentage

*CI* confidence interval 95%

The analysis by age group does not show significant differences with the exception of antisocial personality disorders (*p*-value = 0.038) only present in the age group 30–44 years (3.4%) (as shown in Table 3).

**Table 3:** Lifetime prevalence (%) of DSM-V psychiatric disorders by age groups

Psychiatric disorders	18–29	CI 95%	30–44	CI 95%	45–59	CI 95%	60–74	CI 95%	75+	CI 95%	Totale	CI 95%	Chi-square test	*P*-value
Major depressive episode	19.6	[10.5–33.6]	14.6	[8.7–23.6]	24.6	[17.4–33.5]	25.6	[17.5–35.9]	14.0	[7.2–25.7]	20.4	[16.6–24.7]	1.47	0.21
Major depressive disorder (DDM)	15.2	[7.4–28.7]	10.1	[5.3–18.4]	21.8	[15.1–30.5]	22.1	[14.5–32.1]	12.3	[5.9–23.7]	17.0	[13.6–21.1]	1.84	0.12
Manic episode	4.5	[1.1–16.5]	6.7	[3.0–14.3]	3.7	[1.4–9.4]	3.5	[1.1–10.3]	0.0	–	3.9	[2.4–6.4]	1.08	0.37
Hypomanic episode	4.8	[1.2–17.2]	1.2	[0.2–8.0]	0.0	–	0.0	–	1.8	[0.2–11.5]	1.1	[0.4–2.8]	1.91	0.10
Panic disorder	10.9	[4.6–23.7]	14.6	[8.7–23.6]	9.9	[5.6–17.1]	8.1	[3.9–16.2]	7.0	[2.6–17.3]	10.3	[7.6–13.7]	0.73	0.57
Psychotic disorder	0.0	–	0.0	–	0.0	–	0.0	–	0.0	–	0.0	–	–	–
Antisocial personality disorder	0.0	–	3.4	[1.1–10.1]	0.0	–	0.0	–	0.0	–	0.8	[0.3–2.4]	2.55	0.04
Bipolar I disorder	4.5	[1.1–16.5]	6.7	[3.0–14.3]	3.7	[1.4–9.4]	3.5	[1.1–10.3]	0.0	–	3.9	[2.4–6.4]	1.08	0.37
Bipolar II disorder	0.0	–	0.0	–	0.0	–	0.0	–	1.8	[0.2–11.5]	0.3	[0.0–1.8]	1.45	0.21
At least one of the listed ailments	30.4	[18.9–45.1]	27.8	[19.5–37.9]	30.6	[22.7–39.8]	31.4	[22.5–41.9]	19.3	[11.0–31.6]	28.5	[24.2–33.2]	3.08	0.54

Differences and associations were considered statistically significant if their p-values were less than 0.05

%: percentage

*CI* confidence interval 95%

*In terms of point prevalence*, the results showed that 15.6% of the respondents suffered at that time, or had recently suffered from a mental disorder without significant gender differences (females: 17.6%; males: 13.5%; p-value = 0.271). Specifically, in the last 2 weeks prior to the interview, 3.1% of the sample had experienced a major depressive episode and 2.6% had a major depressive disorder. In the last month, 5.9% had felt the urge to commit suicide or injure themselves and 2.3% had suffered from a social anxiety disorder. In the previous 3 months, 5.4% had suffered from a generalized anxiety disorder. The agoraphobic disorder was currently present in 2.8% of the sample and, over the last year, 2.8% had suffered from an alcohol use disorder. The analysis by gender shows significant differences in females in agoraphobic disorder (*p*-value = 0.009) and in social anxiety disorder (p-value = 0.026) (as shown in table 4).

**Table 4:** Point prevalence (%) of DSM-V psychiatric disorders by gender

Psychiatric disorders	Males		Females		Total			
	Point Prevalence	CI 95%	Point Prevalence	CI 95%	Point Prevalence	CI 95%	*Z*-test	*P*-value
Major depressive episode	1.6	[0.0–3.5]	4.4	[1.6–7.2]	3.1	[1.8–5.4]	−1.58	0.11
Major depressive disorder (DDM)	1.6	[0.0–3.5]	3.4	[0.9–5.9]	2.6	[1.4–4.7]	−1.12	0.26
Suicidality	5.4	[2.2–8.7]	6.4	[3.0–9.8]	5.9	[4.0–8.8]	−0.40	0.69
Suicide behavior disorder	1.1	[0.0–2.6]	1.0	[0.0–2.4]	1.0	[0.4–2.7]	0.10	0.92
Manic episode	0.0	–	0.0	–	0.0	–	–	–
Hypomanic episode	0.0	–	0.0	–	0.0	–	–	–
Panic disorder	0.5	[0.0–1.6]	1.5	[0.0–3.1]	1.0	[0.4–2.7]	−0.90	0.37
Agoraphobia	0.5	[0.0–1.6]	4.9	[1.9–7.9]	2.8	[1.6–5.1]	−2.58	0.01
Social anxiety disorder (social phobia)	0.5	[0.0–1.6]	3.9	[1.2–6.6]	2.3	[1.2–4.4]	−2.21	0.03
Obsessive-compulsive disorder	0.5	[0.0–1.6]	1.5	[0.0–3.1]	1.0	[0.4–2.7]	−0.91	0.36
Posttraumatic stress disorder	0.5	[0.0–1.6]	0.0	–	0.3	[0.0–1.8]	1.06	0.29
Alcohol use disorder	4.3	[1.4–7.3]	1.5	[0.0–3.1]	2.8	[1.6–5.1]	1.71	0.09
Substance use disorder (non-alcohol)	1.6	[0.0–3.5]	0.0	–	0.8	[0.2–2.4]	1.83	0.07
Psychotic disorder	0.0	–	0.0	–	0.0	–	–	–
Anorexia nervosa	0.0	–	0.0	–	0.0	–	–	–
Bulimia nervosa	0.0	–	0.5	[0.0–1.5]	0.3	[0.0–1.8]	−0.96	0.34
Binge-eating disorder	0.0	–	1.5	[0.0–3.2]	0.8	[0.3–2.5]	−1.62	0.10
Geralized anxiety disorder	3.3	[0.7–5.8]	7.4	[3.8–11.0]	5.4	[3.6–8.2]	−1.79	0.07
Bipolar I disorder	0.0	–	0.0	–	0.0	–	–	–
Bipolar II disorder	0.0	–	0.5	[0.0–1.4]	0.3	[0.0–1.8]	−0.95	0.34
At least one of the listed ailments	13.5	[8.6–18.4]	17.6	[12.4–22.8]	15.6	[12.4–19.6]	−1.10	0.27

Differences and associations were considered statistically significant if their p-values were less than 0.05

%: percentage

*CI* confidence interval 95%

Age represents a factor capable of influencing social anxiety disorder (p-value = 0.008) and substance use disorders (p-value = 0.044). In both cases, the most affected age groups are 18–29 years and 60–74 years (as shown in Table 5).

**Table 5:** Point prevalence (%) of DSM-V psychiatric disorders by age groups

Psychiatric disorders	18–29	CI 95%	30–44	CI 95%	45–59	CI 95%	60–74	CI 95%	75+	CI 95%	Totale	CI 95%	Chi-square test	*P*-value
Major depressive episode	2.2	[0.3–14.0]	1.1	[0.2–7.6]	5.5	[2.5–11.7]	3.5	[1.1–10.3]	1.8	[0.2–11.5]	3.1	[1.8–5.4]	0.93	0.45
Major depressive disorder (DDM)	2.2	[0.3–14.0]	0.0	–	5.5	[2.5–11.7]	3.5	[1.1–10.3]	0.0	–	2.6	[1.4–4.7]	1.96	0.01
Suicidality	4.3	[1.1–15.9]	3.4	[1.1–10.1]	4.5	[1.9–10.5]	10.5	[5.5–19.0]	7.0	[2.6–8.8]	5.9	[4.0–8.8]	1.21	0.30
Suicide behavior disorder	0.0	–	0.0	–	0.9	[0.1–6.2]	1.2	[0.2–7.9]	3.6	[0.9–13.3]	1.0	[0.4–2.8]	1.23	0.29
Manic episode	0.0	–	0.0	–	0.0	–	0.0	–	0.0	–	0.0	–	–	–
Hypomanic episode	0.0	–	0.0	–	0.0	–	0.0	–	0.0	–	0.0	–	–	–
Panic disorder	0.0	–	1.1	[0.2–7.8]	1.8	[0.4–7.0]	1.2	[0.1–7.9]	0.0	–	1.0	–	0.43	0.79
Agoraphobia	2.2	[0.3–14.0]	2.3	[0.6–8.7]	1.8	[0.4–7.0]	5.8	[2.4–13.3]	1.8	[0.2–11.5]	2.8	–	0.90	0.46
Social anxiety disorder (social phobia)	4.3	[1.1–15.9]	0.0	–	0.0	–	7.0	[3.2–14.7]	1.8	[0.2–11.7]	2.3	[1.2–4.4]	3.47	0.01
Obsessive-compulsive disorder	2.2	[0.3–14.3]	0.0	–	2.7	[0.9–8.1]	0.0	–	0.0	–	1.0	[0.4–2.7]	1.51	0.19
Posttraumatic stress disorder	0.0	–	1.1	[0.2–7.6]	0.0	–	0.0	–	0.0	–	0.3	[0.0–1.8]	0.84	0.50
Alcohol use disorder	4.3	[1.1–15.9]	5.7	[2.4–13.0]	2.7	[0.9–8.1]	0.0	–	1.8	[0.2–11.5]	2.8	[1.6–5.1]	1.44	0.22
Substance use disorder (non-alcohol)	4.3	[1.1–15.9]	0.0	–	0.0	–	1.2	[0.2–7.9]	0.0	–	0.8	[0.2–2.4]	2.45	0.04
Psychotic disorder	0.0	–	0.0	–	0.0	–	0.0	–	0.0	–	0.0	–	–	–
Anorexia nervosa	0.0	–	0.0	–	0.0	–	0.0	–	0.0	–	0.0	–	–	–
Bulimia nervosa	0.0	–	0.0	–	0.0	–	1.2	[0.2–7.9]	0.0	–	0.3	[0.0–1.8]	0.89	0.47
Binge-eating disorder	2.5	[0.3–15.9]	2.4	[0.6–9.0]	0.0	–	0.0	–	0.0	–	0.8	[0.3–2.5]	1.46	0.21
Generalized anxiety disorder	6.5	[2.1–18.4]	4.5	[1.7–11.4]	7.3	[3.7–14.0]	4.7	[1.7–11.8]	3.5	[0.9–13.0]	5.4	[3.6–8.2]	0.39	0.82
Bipolar I disorder	0.0	–	0.0	–	0.0	–	0.0	–	0.0	–	0.0	–	–	–
Bipolar II disorder	0.0	–	0.0	–	0.0	–	0.0	–	1.8	[0.2–11.5]	0.3	[0.0–1.8]	1.45	0.21
At least one of the listed ailments	19.6	[10.5–33.6]	15.6	[9.4–24.6]	14.4	[9.0–22.3]	19.8	[12.6–29.6]	8.8	[3.7–19.4]	15.6	[12.3–19.6]	3.81	0.43

Differences and associations were considered statistically significant if their p-values were less than 0.05

%: percentage

*CI* confidence interval 95%

We did not find any statistically significant difference by educational level.

## Discussion

According to our findings, 28.5% of the sample suffered from a mental disorder during their lifetime. This prevalence is considerably higher than those from a previous study in central Italy [10], which highlighted a 24.4% rate of psychiatric disorders during lifetime. A trend towards a higher prevalence of mental disorders among females (33.2%) than among males (29.7%) was detectable, although without statistical significance. This result is in line with previous researches that reported a statistically significant higher prevalence of mental disorders among females in Italy and in other Countries [10, 15]. Recent literature stressed the need to rethink the issue of gender differences in psychiatric disorders in light of cultural and environmental biases, which may lead to underestimate psychiatric disorders among men. One of these biases could be identified in males’ reticence to speak about their mental health problems [16, 17]. In this framework, it is possible that, among males, only subjects more interested in speaking about this topic agreed to participate in the interviews. Furthermore, some authors highlighted a greater tendency of participants to reveal their symptoms during phone interviews, compared to face-to-face ones [18, 19]. The protocol followed in our study, avoiding direct contact, might have facilitated the expression of symptoms by male participants [16, 17].

In our sample, the most frequent lifetime mental disorder was major depressive disorder (17.0%), while the lifetime prevalence of major depressive episodes was 20.4%. This result is in line with previous studies reporting major depressive disorder or major depressive episodes as the disorders with the highest lifetime prevalence [10, 20]. While Kessler et al. [20] reported a similar lifetime prevalence of major depressive disorder in the USA, the lifetime prevalence of major depressive episodes in the study by Faravelli et al. [10], conducted in central Italy, was lower (9.5%) than the prevalence reported here. Considering bipolar disorders, the lifetime prevalence of bipolar I-II disorders was similar to that reported by Kessler et al. [20], but higher than the one reported by Faravelli et al. [21]. The differences seem to be mainly ascribable to bipolar I disorder, for which we highlighted a lifetime prevalence of 3.9%, which is higher than those reported in other epidemiological studies in Italy and other Countries [21, 22]. This discrepancy is in line with the growing evidences indicating that the prevalence of bipolar disorder in the general population might be underestimated [23]. Smaller differences with previous results were found for bipolar II disorder [21, 22]. It should be noted that the presence of hypomanic episodes during lifetime could be difficult to detect in one single interview assessment [10, 21]; as a result, the prevalence of bipolar II disorder could have been underestimated.

The current prevalence of major depressive episodes reported here confirmed the rates from a previous study in central Italy [24]. The current prevalence of major depressive disorder was in agreement with the current prevalence reported in other Countries, which ranges between 0.9 and 5.6% [25]. Faravelli et al. [24] reported a current prevalence of manic/hypomanic episodes of 0.3%, which could be in line with the absence of current manic/hypomanic cases reported here.

In the field of anxiety disorders, our data only reported lifetime prevalence for panic disorder. Generalized anxiety disorder showed the highest current prevalence (5.4%) among anxiety disorders, followed by agoraphobia (2.8%) and social anxiety (2.3%). In previous studies, the most frequent anxiety disorders were generalised anxiety disorder and social phobia, although with a high variability of rates [10, 20, 24]. In our sample, the current rates of anxiety disorders were higher than those observed in the previous study carried out in central Italy, which considered both current and one-year prevalence [24]. The lifetime panic disorder rate was also increased when compared with previous findings and with the prevalence rates reported in DSM-5 [10, 20, 24, 26].

The prevalence of current feeding and eating disorders was similar to those from previous studies [24, 27], although we did not find any case of restrictive anorexia nervosa. It should be noted that anorexia prevalence is generally lower than 0.5%, and the absence of cases in a sample of 390 subjects could be in line with previous findings. Moreover, the MINI considers a Body Mass Index (BMI) equal to or less than 17 kg/m^2^ as a threshold for the diagnosis of anorexia nervosa, whereas the recommended cut-off for identifying underweight in clinical and research settings is 18.5 kg/m^2^ [28, 29]: this led to the exclusion of all “mild” cases of anorexia nervosa according to DSM-5 (BMI ≥ 17.0 kg/m^2^) [26]. Considering non-affective psychoses, our data, showing a lack of cases, could be in agreement with the 0.7% lifetime rate from previous findings [10]. However, it could also be possible that subjects with schizophrenia spectrum disorders, even if contacted, refused to participate or not answered phone invitations.

In line with previous literature, we found a significantly higher prevalence of current agoraphobia and social phobia, as well as of lifetime major depressive disorder and major depressive episodes, among females, although the prevalence of lifetime major depressive disorder in both genders was higher than the ones previously reported in central Italy [10]. A trend toward the increased prevalence of current major depressive disorders and major depressive episodes among females was reported, although the statistical significance was not reached. A non-significant trend towards higher rates among females was also reported for lifetime and current panic disorder, whose prevalence was found to be strikingly increased when compared with previous findings [10, 20]. The rates of current Post-Traumatic Stress Disorder (PTSD) in our sample were similar to those reported before the pandemic [24, 30–32]. While other studies conducted after the spread of COVID-19 reported increased rates of PTSD [33, 34], our work was conducted at the beginning of the COVID-19 outbreak and during the first lockdown: thus, even if some of the subjects developed a PTSD related to the COVID-19 pandemic, at the time of the interview an insufficient amount of time had passed for receiving a PTSD diagnosis according to DSM-5 [26].

According to other studies, we found a higher prevalence among males for alcohol use disorder while no cases of other substance use disorders were found among females. The overall prevalence in our sample of alcohol/other substance use disorders in the last year was higher than the 0.2% reported by Faravelli et al. [24], but lower than the prevalence reported in the USA (about 8–12% for alcohol use disorder and 2–3% for illicit drugs) [27, 32]. However, as noted by other authors [24], the eventual lower prevalence of alcohol/substance use disorders in Italy should be considered with extreme caution.

Finally, the prevalence of subjects who showed suicidal ideation in the last month or suicidal behaviour in the last 12 months is in line with previous studies in Europe, as reported by a recent meta-analytic review [35]. However, these results should be considered in light of the high heterogeneity of the methods used for assessing these conditions [35].

Considering age group analysis**,** a significant difference was found for antisocial personality disorder, for which all the recorded cases were in the age group of 30–44 years. Even if lifetime prevalence of anxiety and mood disorders should be expected to increase with age, it is possible that other factors could be accountable for the lack of age differences in this study, including eventual different approaches towards phone interviews depending on age or the older people’s tendency to under-report symptoms [36]. In this framework, it is noteworthy that Kessler et al. [20], despite reporting that lifetime prevalence increased with age for several disorders, highlighted a decrease in the oldest age range.

## Strengths and limitations

This work has several limitations. First, the study was conducted in a relatively small sample. In this framework, some results, such as the absence of statistical significance for gender differences reported in psychiatric disorders prevalence should be evaluated in light of an eventual low statistical power. Secondly, the study was led in a specific regional area, and our results may not be extensible to other Italian regions or Countries. Moreover, the study was based on a single instrument, and the interviews were carried out by phone, without any external confirmation of subjects’ statements, eventually leading to biases in the assessment of symptoms. The specific recruitment method, carried out by phone, might have also led to selection biases, eventually facilitating the participation of some subjects (e.g. people more interested in the topic) or the lack of inclusion of specific populations (e.g. schizophrenic patients). In addition, the version of the MINI used in this study does not assess the lifetime prevalence of some disorders, including obsessive-compulsive disorders, anxiety disorders (except panic disorder), and alcohol or substance use disorders, and does not consider common disorders such as specific phobias, persistent depressive disorder, body dysmorphic disorder, personality disorders (except antisocial personality disorder), and others; consequently, the total prevalence of both lifetime and current mental disorders was most likely underestimated.

In the evaluation of results from this study, it should be noted that the prevalence rates of mental disorders reported in previous works showed high variability, probably depending on the assessment methods, and that the prevalence rates of overall psychiatric disorders and mood disorders in our sample were similar to those reported in other countries [20, 37]. However, our data highlighted a higher prevalence of mental disorders, in particular lifetime mood disorders and panic disorder, as well as current anxiety disorders, when compared to previous studies in central Italy [10, 21]. In this study, all interviews were administered by psychiatry residents and not by general practitioners as in previous Italian researches [10, 21]; this may partly explain the discrepancy between the observed prevalence, considering that a concordance between MINI diagnoses obtained by general practitioners and experienced specialists was observed in only 85% of patients [12]. In addition, the phone interview may have facilitated the expression of symptoms among more reticent subjects [16, 17]. However, our data seem in line with recent works that stressed an increase of anxiety disorders after the spread of COVID-19 [33, 34, 38–40]. The specific pandemic environment in which the interviews were performed might at least partially explain the increased rates of mental disorders with respect to previous studies in the same area [41]. Similarly, results from our study may be in line with the recorded increase of mental health problems in different European Countries after the global economic crisis of 2008–2010 [42].

## Conclusion

Our findings provide updated data about the prevalence of mental disorders in the investigated area, showing a significant increase in the prevalence of mental disorders in the general adult population, particularly for depressive disorder. These results could open the way to further research in the field, which may allow a better understanding of the variables involved in mental disorder prevalence’s changes over time. In addition, our findings may help clinicians in tailoring more targeted prevention, detection and treatment strategies for mental health, in order to address the increase of mental disorders in the general population.

## Supplementary Information

**Additional file 1 Supplementary Table 1.** Distribution of the Educational Level by gender

## Data Availability

The datasets used and/or analysed during the current study are available from the corresponding author upon reasonable request.

## Abbreviations

M.I.N.I: Mini-International Neuropsychiatric Interview
WHO: World Health Organization
ESEMeD: European Study of the Epidemiology of Mental Disorders
WMH: World Mental Health
LHU: Local Health Units
DSM-5: Diagnostic and Statistical Manual of mental disorders V
ICD 10: International Classification of Diseases 10
SCID-P: Structured Clinical Interview for DSM-III-R- Patient
CIDI: Composite International Diagnostic Interview
GAD: Generalised Anxiety Disorder
CI: Confidence Interval
χ2: Chi-square
F: tests or Fisher’s
BMI: Body Mass Index
PTSD: Post-Traumatic Stress Disorder
