# Supplementary material for: Prevalence study of mental disorders in an Italian region. Preliminary report
Source: BMC Psychiatry. 2023 Jan 5;23:12. doi: 10.1186/s12888-022-04401-4 (PMC9812746; doi:10.1186/s12888-022-04401-4)
Supplement: Supplementary file 1 — Additional file 1 Supplementary Table 1. Distribution of the Educational Level by gender [file 12888_2022_4401_MOESM1_ESM.docx]

**Supplementary table 1: Distribution of the Educational Level by gender**

| **Educational Level** | **Males** | | **Females** | | **Total** | |
| --- | --- | --- | --- | --- | --- | --- |
|  | **N** | **%** | **N** | **%** | **N** | **%** |
| Low | 18 | 11.9 | 28 | 17.2 | 46 | 14.6 |
| Medium | 98 | 64.9 | 92 | 56.4 | 190 | 60.5 |
| High | 35 | 23.2 | 43 | 26.4 | 78 | 24.8 |
| **Total** | 151 | 100.0 | 163 | 100.0 | 314 | 100.0 |

Education level: low ≥ 5 and 8 years); medium > 8 and 13 years; high > 13 years.

N: number

%: percentage
